# Outcomes by Race in Breast Cancer Screening With Digital Breast Tomosynthesis Versus Digital Mammography

**DOI:** 10.1016/j.jacr.2020.12.033

**Published:** 2021-02-17

**Authors:** Nila Alsheik, Linda Blount, Qiu Qiong, Melinda Talley, Scott Pohlman, Kathleen Troeger, Genevieve Abbey, Victoria L. Mango, Erica Pollack, Alice Chong, Greg Donadio, Michael Behling, Kathleen Mortimer, Emily Conant

**Affiliations:** a Section Chief, Division Breast Imaging. Co-Medical Director, Advocate Caldwell Breast Center; Chair, Advocate Breast Imaging Medical Directors Committee; Chair, AAHC Imaging Research Council; Advocate Lutheran General Hospital, Park Ridge, Illinois.; b President and CEO of Black Women’s Health Imperative; Vice-Chair of the board of Creating Healthier Communities; Chair of the Rare Disease Diversity Coalition, Washington, District of Columbia.; c Advocate Caldwell Breast Center, Park Ridge, Illinois.; d Sanford Health, Sioux Falls, South Dakota.; e Director, Outcomes Research at Hologic, Marlborough, Massachusetts.; f Senior Director, Outcomes Research at Hologic, Marlborough, Massachusetts.; g Program Manager, RAD-AID Breast Imaging, RAD-AID International.; h Weill Cornell Medicine/NewYork-Presbyterian Hospital, New York, New York.; i Director of Radiology at the Ralph Lauren Center for Cancer Care at Memorial Sloan Kettering; Head of Breast Imaging Clinical Research at Memorial Sloan Kettering; Associate Training Program Director Breast Imaging Fellowship at Memorial Sloan Kettering, Memorial Sloan Kettering Cancer Center, New York, New York.; j Director, RAD-AID Kenya, Nairobi, Kenya.; k Director of Breast Imaging at RAD-AID, RAD-AID International.; l Head of the breast imaging division at Denver Health and Hospital Authority, Denver, Colorado.; m Associate Professor, Department of Radiology, University of California, San Diego, La Jolla, California.; n Vice Chair of Clinical Operations, Department of Radiology, RAD-AID Breast Imaging Program Manager, RAD-AID International.; o OM1, Boston, Massachusetts.; p Division Chief, Breast Imaging Hospital of the University of Pennsylvania Vice Chair of Faculty Development, Department of Radiology, Perelman School of Medicine, Professor of Radiology at the Perelman School of Medicine, University of Pennsylvania, Philadelphia, Pennsylvania.

**Keywords:** Breast cancer screening, racial disparities, digital breast tomosynthesis

## Abstract

**Purpose:**

Digital breast tomosynthesis (DBT) in conjunction with digital mammography (DM) is becoming the preferred imaging modality for breast cancer screening compared with DM alone, on the basis of improved recall rates (RR) and cancer detection rates (CDRs). The aim of this study was to investigate racial differences in the utilization and performance of screening modality.

**Methods:**

Retrospective data from 63 US breast imaging facilities from 2015 to 2019 were reviewed. Screening outcomes were linked to cancer registries. RR, CDR per 1,000 examinations, and positive predictive value for recall (cancers/recalled patients) were compared.

**Results:**

A total of 385,503 women contributed 542,945 DBT and 261,359 DM screens. A lower proportion of screenings for Black women were performed using DBT plus DM (referred to as DBT) (44% for Black, 48% for other, 63% for Asian, and 61% for White). Non-White women were less likely to undergo more than one mammographic examination. RRs were lower for DBT among all women (8.74 versus 10.06, *P* < .05) and lower across all races and within age categories. RRs were significantly higher for women with only one mammogram. CDRs were similar or higher in women undergoing DBT compared with DM, overall (4.73 versus 4.60, adjusted *P* = .0005) and by age and race. Positive predictive value for recall was greater for DBT overall (5.29 versus 4.45, adjusted *P* < .0001) and by age, race, and screening frequency.

**Conclusions:**

All racial groups had improved outcomes with DBT screening, but disparities were observed in DBT utilization. These data suggest that reducing inequities in DBT utilization may improve the effectiveness of breast cancer screening.

## Graphical Abstract

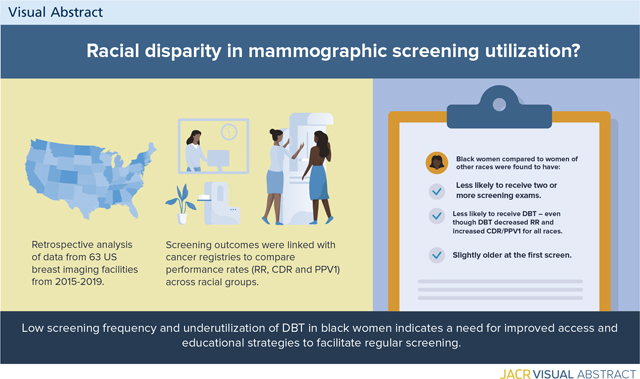

## INTRODUCTION

The United States has one of the highest age-standardized incidence rates of breast cancer in the world (72.9 per 100,000) [1]. Excluding skin cancers, breast cancer continues to be the most commonly diagnosed neoplasm for women, with 268,000 new cases of invasive cancer diagnosed in 2019 [2]. Early detection of breast cancer has been facilitated by widespread access to mammographic screening, which has undergone significant evolution since first implemented in the 1960s. By the early 2000s, full-field digital mammography (DM) had replaced analog film-screen mammography [3,4]. As two-dimensional imaging modalities, however, film-screen mammography and DM have limited overall sensitivity in detecting breast cancers, especially in dense, fibroglandular breast tissue [5,6]. In 2011, digital breast tomosynthesis (DBT) received US Food and Drug Administration approval. DBT generates a quasi-three-dimensional mammogram by obtaining multiple low-dose exposures across a limited arc, which are then reconstructed into a series of images or “slices” of the breast. Population-based studies from both the United States and Europe have demonstrated initial and sustained reductions in recall rates (RR) and/or increases in invasive cancer detection rates (CDRs) with DBT plus DM (referred to as DBT throughout) compared with DM screening alone [7–10].

Advances in screening and treatment of breast cancer have led to a steady decline of breast cancer–related mortality in the past 15 years, with an overall 5-year relative survival rate of 89% [11]. These advances, however, have not benefited women equally across all ages, socioeconomic backgrounds, geographic regions, and races [12,13]. Black women are more likely to be diagnosed with breast cancer at a younger age and more advanced stage and to die of breast cancer [14]. As a result, breast cancer carries one of the highest observed racial disparities in mortality and 5-year relative survival rates between White and Black women, despite similar diagnosis rates, a disparity that is growing [11,15]. Increased rates of advanced-stage cancer at diagnosis are driven by delayed diagnoses resulting from barriers to mammographic screening, as well as higher rates of aggressive, poor prognostic, triple-negative breast cancers among Black women [11,16,17]. These disparities may be amplified by variations in the adoption and dissemination of new technology [18]. The adoption of DBT has been faster in areas with higher median incomes and larger proportions of White residents [19]. Understanding the epidemiologic impact of varied adoption rates for new technologies such as DBT on racial disparities in breast cancer is therefore needed. Although the performance of DBT has been well studied in general screening populations, there is limited evaluation of the performance of DBT within racial groups. The aim of this study was to understand the impact of DBT on health disparities by evaluating the use of DBT within three large health systems and the impact on performance metrics such as RR, CDR, and positive predictive value (PPV1) across racial groups.

## METHODS

Screening mammograms were identified from radiology databases at three large health care systems from January 2015 to January 2019 using screening code descriptors corresponding to bilateral asymptomatic screening mammograms. The analysis was restricted to women without histories of cancer or breast implantation. Given the focus on racial disparities, only women with reported race information in their medical records were included. This study was HIPAA compliant and received approval from the institutional review boards at the three participating health care delivery organizations throughout metropolitan Chicago (AdvocateAurora Health Care), the greater Philadelphia area (University of Pennsylvania), and South Dakota and surrounding area (Sanford Health).

Characteristics of the screened population include selfreported race (Black, White, Asian, or other), ethnicity (Hispanic, non-Hispanic, or unknown), age at the first screen observed in the study period, menopausal status (recorded by the site or set to postmenopausal if status was missing and age was >59 years), breast density (almost entirely fatty, scattered fibroglandular densities, heterogeneously dense, extremely dense, or unknown), number of observed screens during the study period, screening modality (DM or DBT), and institution. For patients at two institutions, the 5-year risk Gail model was used, and scores ≥1.66 were considered to indicate elevated risk. For patients at the other site, a lifetime Tyrer–Cuzick risk score of ≥20% was considered elevated risk.

Recall was defined as a BI-RADS assessment category of 0 (incomplete test, need for additional imaging), 4 (suspicious findings or abnormalities), or 5 (highly suspicious findings) on a screening examination or at a recall from a recent screening examination. RR was calculated as the number of recalled screens divided by the number of screens with recorded BI-RADS scores. RRs were stratified by race, age, screening modality, and screening frequency. Overall P values were calculated using the χ^2^ test or analysis of variance for the comparison of DBT and DM outcomes. Odds ratios (ORs) for recall (1 = recall, 0 = no recall) and 95% confidence intervals (CIs) were calculated by race and screening modality using logistic regression with adjustments for age, institution, and breast density.

Screening data were linked to a state cancer registry (one site) and hospital-level tumor registries (two sites) using internal institutional patient identifiers. CDR per 1,000 screens (number of cancers/number of women screened × 1,000) was reported by modality overall and separately by age and race. PPV1 (number of cancers/number of screens with recalls) was reported for DBT versus DM overall and separately by age group, race, and screening frequency. The CDR and PPV1 analyses were restricted to time periods when the tumor registry data were expected to be complete (screening dates from July 2015 to June 2018).

## RESULTS

Data included women screened at 63 imaging facilities across the institutions. After excluding 12,315 women without race information, 804,304 screening mammograms (542,945 DBT, 261,359 DM) from 385,503 women were included (Table 1). Among the 63 facilities, 12 did not perform DBT imaging, and four did not perform DMonly imaging. Given the low percentage of Hispanic women and missing data on ethnicity, results focus on racial rather than ethnic comparisons. More White women had two or more screens during the study period compared with women of other races (63.7% for White, 57.0% for Blacks, 51.6% for Asians, and 49.6% for other races). Black women were significantly less likely to have two or more screenings relative to White women after adjustment for age and institution (OR, 0.895; 95% CI, 0.881–0.909; *P* < .0001; data not shown). Demographic and screening utilization data by race are summarized for all women screened (Table 2). The overall mean age at the first screen within the study period was 57.0 years, with slightly higher mean ages at the first screen for White (57.2 years) and Black women (57.3 years) compared with Asian women (55.2 years) and women of other races (54.3 years) (*P* < .001). Among those with reported breast density, a higher proportion of Asian women had heterogeneously dense or extremely dense breasts (68.3%) compared with White (49.2%) or Black women (40.1%). Asian women (63.1%) and White women (60.5%) were more likely to have at least one DBT screen compared with Black women (44.4%) (*P* < .001). The majority of Black, Asian, and other race women were screened at two of the sites, whereas the population from the third site was predominantly White. Among women with reported risk scores (74.3%), a higher percentage of White women were defined as having elevated risk compared with women of other races (White, 11.2%; Black, 5.6%; Asian, 5.1%; *P* < .001).

Overall, the aggregate RR was 8.74% for DBT compared with 10.06% for DM screening (*P* < .0001; Table 3). RRs were significantly lower for DBT versus DM across all races and age categories after adjusting for breast density and institution. The absolute RR reduction associated with DBT was most substantial for Asian women (2.43%), followed by Black women (2.02%) and White women (1.00%). Recall reduction was most pronounced in the 40- to 49-year age category for all races except for Asian women.

RRs were significantly higher among the 150,035 women (38.9%) with only one screen compared with the 235,468 women (61.0%) with two or more screens during the study period (DM, 8.14% versus 18.03%; DBT, 7.26% versus 15.35%; *P* < .0001 for both). RRs were lower with DBT versus DM for women with one screen (Table 4) and two or more screens (Table 5). Among women with one screen, the largest absolute recall reduction associated with DBT was 4.02% (Asian), followed by 3.45% (Black) and 2.53% (White), with a 2.68% reduction overall (Table 4). Among women with at least two screens, the largest absolute recall reduction associated with DBT was 1.84% (Black), followed by 1.42% (Asian) and 0.54% (White), with a 0.88% reduction overall (Table 5).

DBT was associated with a statistically significant reduction in recalls compared with DM (OR, 0.870; 95% CI, 0.856–0.885), adjusted for age, institution, and breast density (Table 6). The lower odds of recalls with DBT versus DM were most notable among Black women (OR, 0.813; 95% CI, 0.785–0.841) and Asian women (OR, 0.817; 95% CI, 0.756–0.881) but was also significant among White women (OR, 0.897; 95% CI, 0.879–0.916).

During the period with complete cancer registry data (screening from July 2015 to June 2018), 2,339 breast cancers were diagnosed among 499,376 eligible screens (4.68 per 1,000). Overall, the CDR was slightly higher among women screened with DBT compared with DM (4.73 versus 4.60 per 1,000 screens, adjusted *P* = .0005; Table 7). DBT resulted in similar or higher CDRs per 1,000 screens by age group: 40 to 45 years, 2.30 versus 1.90; 50 to 59 years, 4.28 versus 3.83; 60 to 69 years, 5.71 versus 5.51; 70 to 79 years, 7.23 versus 6.84; with the exception of women aged 46 to 49 years (2.91 versus 3.13) (*P* > 0.05 for all). CDRs increased with age; the highest CDR was in the 70- to 79-year age group, regardless of imaging modality (7.23 versus 6.84 per 1,000 screens for DBT versus DM, respectively). CDRs associated with DBT were higher than those for DM in the two largest represented racial groups, White women (4.79 with DBT versus 4.62 with DM, *P* = .0016) and Black women (4.89 with DBT versus 4.76 with DM, *P* = .2083).

PPV1 was greater for DBT compared with DM for the cohort overall (5.29 versus 4.45, adjusted *P* < .0001) and across age group, race, screening frequency, and breast density (Table 8). The magnitude of differences for PPV1 with DBT compared with DM consistently increased from the youngest age group (0.28) to the oldest age group (2.16). The largest improvement in PPV1 by race was among Black women, with a PPV1 of 5.48 (DBT) versus 4.42 (DM).

## DISCUSSION

On the basis of data from 63 US breast imaging facilities, racial differences in the modality and frequency of breast cancer screening were found. Relative to White women, a smaller proportion of Black women had multiple screens, and a lower proportion underwent DBT. However, Black women who underwent DBT had lower RRs than White women. Asian women also had lower proportions of women with multiple screens and higher RRs but a larger proportion of DBT compared with DM.

This study demonstrated that relative to DM alone, the addition of DBT for breast cancer screening is associated with improved patient screening metrics, including RR, CDR, and PPV1, for women of all ages and races, although not all comparisons reached statistical significance. Improvements are consistent with those seen in other studies that analyzed performance in aggregate but did not report results by racial group. [7–9] Studies that quantify racial disparities are instrumental to the identification and implementation of customized solutions to improve breast cancer outcomes in select populations.

This study demonstrated that more frequent screening was associated with lower RR, for both DBT and DM, as has been described previously [7–10]. Although the observed overall RR was within the ACR’s recommended range of 5% to 12% [20], RR varied widely by subpopulation, from a low of 6.70% in Black women with at least two screenings and screened with DBT to a maximum of 20.98% in Asian women screened once with DM. This latter finding for Asian women may result from the combination of longer screening intervals as well as the larger proportion of women with higher breast density, both of which influence the rate of screening recall [21].

Some of the observed racial differences in screening frequency and modality may be due to variations in actual and perceived baseline risk. Specifically, more White women met the definition of high risk and were likely referred for more frequent or supplemental screening with additional imaging modalities. Awareness of increased risk scores may have resulted in White women’s prioritizing recommended screening timelines or influenced physicians’ referral patterns. However, despite having twice the likelihood of being considered high risk, CDRs are similar for White and Black women by screening modality. Given the evidence that these risk scores may underestimate the risk in Black women, use of these scores to influence recommended screening intervals and modalities should be undertaken with caution [22]. Future research is needed to explore racial differences in risk assessment through development of comprehensive electronic medical record–based risk scores.

Racial differences in screening frequency and DBT utilization are likely rooted in social, economic, cultural, and educational disparities [23]. Less frequent screening of Black women indicates a need for improved access and educational strategies to emphasize the importance of regular screening. Harvey et al [24] reported that interactions with primary care providers significantly influence screening utilization. Thus, if providers are educated about the benefits of more frequent screening, applicability of risk scores for across racial groups, and the improved screening outcomes achieved with DBT, they may promote DBT uptake by their patients, potentially resulting in increased DBT access. Knowledge of DBT benefits may also lead to patient-initiated requests for DBT availability and screening, but racial differences in inadequate access to primary care must be considered.

Although challenging to address, interventions that increase awareness and enable primary care and breast imaging providers to decrease barriers to screening, particularly DBT, could have a positive impact. Expanded access to DBT and the opportunity for earlier diagnosis through improved cancer detection are especially relevant for Black women because of increased late-stage diagnoses and resulting decreased breast cancer survival rates in this population [11,14,15]. A recent study comparing DM with DBT screening outcomes over a 5-year period, in a racially diverse population with approximately 50% Black women, showed that DBT at both first and subsequent screening identified more cancers with poor prognosis than those detected by DM [10]. In addition, there was a trend toward decreasing false-negative results with DBT compared with DM.

Reductions in RR occurred in parallel with increases in CDR and reductions in false-positive findings. Benefits of improvements in these screening metrics likely include decreases in patient stress, time away from work, cost for diagnostic imaging, and the number of biopsies with benign results. Previous reports suggest that the average cost of a recall from a commercial payer perspective is $1,200 [25]. Unnecessary recalls may also result in copays directly for patients; although the Patient Protection and Affordable Care Act mandates that women cannot be charged a copay for screening, that is not the case for diagnostic imaging or biopsies [26]. Costs associated with false-positive screenings impose a greater burden on women in lower socioeconomic groups and must be considered in further evaluation of breast cancer racial disparities.

Richman et al [19] showed that regions with slow DBT adoption had lower median household incomes and higher percentages of African Americans than regions with faster DBT adoption, suggesting that inconsistent adoption of DBT may play a significant role in the disparities identified. Thus, institutions should consider potential ramifications of incomplete adoption of DBT on racial disparities within their networks. Advances in electronic medical record–based scheduling systems in regions with underserved populations have been proposed to address racial, economic and other disparities [27].

Barriers to adequate screening vary by race, insurance status, and income and include access to transportation, child and elder care, inability to obtain time away from work, and cost. In particular, previous research on psychosocial factors influencing Black women’s decisions to screen reveals barriers due to distrust of the health care system, fear, fatalistic perceptions of cancer, inaccurate perceptions of risk, and associations with stigma [14]. Data suggest that false-positive results in Black women adversely affect subsequent screening rates [28]. Insurance coverage affects care decisions, and during this study period, insurance coverage of DBT was incomplete. In some cases, DBT screening required additional out-of-pocket payments. Although this study does not include an assessment of payment by race, information on the additional cost for DBT was reported by each of the three centers. At AdvocateAurora Health (more than 50% of patients), there was no additional charge for DM versus DBT. At UPenn, there was initially a larger charge for DBT, but this disparity was ended by July 2016. At Sanford, patients received brochures regarding possible institutional foundation support for additional DBT-related screening.

Clinical guidelines affect the uniform adoption of DBT across racial groups. For example, the ACR, the National Comprehensive Cancer Network, and the American Society of Breast Surgeons include DBT in their screening guidelines, whereas other organizations, such as the US Preventive Services Task Force and the American College of Obstetricians and Gynecologists, do not. Additionally, these organizations provide conflicting guidelines regarding screening frequency. Real-world data such as those from this study can be used in efforts for guideline improvement.

There were several study limitations. The results from the three US health systems may not represent national practice or global performance. Asian and Hispanic women were underrepresented compared with national demographics. The study design did not allow the determination of whether women had been screened at study facilities before the study period or had been screened elsewhere. Lack of income and insurance status data limited the analysis of the impact of socioeconomic status or health care insurance coverage on access and utilization, and some observed disparities across racial groups may be confounded by socioeconomic status. Not all facilities completed both DBT and DM screening, which may have confounded issues of access. Last, cancer registry reporting was needed to calculate CDR and PPV1, and because of the lag for case reporting to these registries, case ascertainment may not have been complete. The study population for the cancer analyses is therefore more restricted than for the RR analysis, leading to reduced statistical power.

Although disparities in DBT utilization were identified, this study was not designed to fully investigate all the underlying reasons for these disparities. Additional research is required to elucidate these causes. It is unlikely that women can entirely influence their screening modalities, and therefore, interventions at societal, facility, and provider levels to ensure appropriate access to DBT are warranted.

## CONCLUSIONS

Racial disparities in mammographic screening utilization were identified overall and specifically for DBT screening. Although not all comparisons reached statistical significance, this study suggests that that the addition of DBT screening to DM is associated with improved screening performance, including improved RR, CDR, and PPV1 across all racial groups. Therefore, these data suggest that overcoming the existing disparities in DBT utilization may be key to improvement in the effectiveness and equity of breast cancer screening.

## Figures and Tables

**Table 1.** Racial distribution of screened women

	Women With ≥2 Screens (n = 235,468)		Women With 1 Screen (n = 150,035)		
Characteristic	n	%	n	%	P

Race					
Black	47,057	20.0	35,465	23.6	<.0001*
White	170,853	72.6	97,456	65.0	<.0001*
Asian	9,269	3.9	8,692	5.8	<.0001*
Other	8,289	3.5	8,422	5.6	<.0001*
Ethnicity					
Hispanic	13,094	5.9	9,522	7.9	<.0001*
Non-Hispanic	208,827	94.1	111,359	92.1	<.0001*
Unknown	13,547		29,154		<.0001*

* *P* < .05 for pairwise tests for the distribution of races between 1 screen and 2 screens.

**Table 2.** Characteristics of the screened population at first screen, by race

	Race					
	White (n = 268,309)	Black (n = 82,522)	Asian (n = 17,961)	Other (n = 16,711)	Total (n = 385,503)	P

Age (y)						<.001*
n	268,309	82,522	17,961	16,711	385,503	
Mean ± SD	57.2 ± 10.2	57.3 ± 10.3	55.2 ± 10.2	54.3 ± 10.0	57.0 ± 10.2	
Median (IQR)	57 (49–65)	57 (49–65)	54 (46–63)	53 (46–62)	57 (49–65)	
Age categories						<.001^†^
40–44 y	35,615 (13.3)	10,541 (12.8)	3,299 (18.4)	3,379 (20.2)	52,834 (13.7)	
45–49 y	35,817 (13.3)	11,239 (13.6)	3,116 (17.3)	3,023 (18.1)	53,195 (13.8)	
50–59 y	85,797 (32.0)	26,158 (31.7)	5,281 (29.4)	5,257 (31.5)	122,493 (31.8)	
60–69 y	74,956 (27.9)	22,751 (27.6)	4,426 (24.6)	3,546 (21.2)	105,679 (27.4)	
70–79 y	36,124 (13.5)	11,833 (14.3)	1,839 (10.2)	1,506 (9.0)	51,302 (13.3)	
Menopause status						<.001^†^
Postmenopausal	128,660 (70.2)	42,380 (76.4)	7,822 (72.4)	6,500 (74.5)	185,362 (71.8)	
Premenopausal	54,518 (29.8)	13,118 (23.6)	2,989 (27.6)	2,223 (25.5)	72,848 (28.2)	
Unknown	85,131	27,024	7,150	7,988	127,293	
Lifetime risk status^‡^						<.001^†^
Average	163,216 (88.8)	70,894 (94.4)	13,506 (94.9)	12,595 (94.1)	260,211 (90.8)	
Elevated	20,616 (11.2)	4,217 (5.6)	719 (5.1)	783 (5.9)	26,335 (9.2)	
Unknown	84,477	7,411	3,736	3,333	98,957	
Breast density						<.001^†^
Almost entirely fatty (A)	17,736 (6.9)	7,657 (9.3)	389 (2.2)	828 (5.0)	26,610 (7.1)	
Scattered fibroglandular densities (B)	113,240 (43.9)	41,695 (50.6)	5,285 (29.6)	7,205 (43.4)	167,425 (44.6)	
Heterogeneously dense (C)	103,812 (40.2)	29,605 (35.9)	9,393 (52.6)	7,304 (44.0)	150,114 (40.0)	
Extremely dense (D)	23,277 (9.0)	3,496 (4.2)	2,798 (15.7)	1,265 (7.6)	30,836 (8.2)	
Unknown	10,244	69	96	109	10,518	
Number of screens during study period						<.001^†^
1	97,456 (36.3)	35,465 (43.0)	8,692 (48.4)	8,422 (50.4)	150,035 (38.9)	
≥2	170,853 (63.7)	47,057 (57.0)	9,269 (51.6)	8,289 (49.6)	235,468 (61.1)	
Screening modality (first screen)						<.001^†^
DM	105,871 (39.5)	45,883 (55.6)	6,623 (36.9)	8,701 (52.1)	167,078 (43.3)	
DBT	162,438 (60.5)	36,639 (44.4)	11,338 (63.1)	8,010 (47.9)	218,425 (56.7)	
Site						<.001^†^
AdvocateAurora Health	144,442 (53.8)	55,265 (67.0)	12,752 (71.0)	12,664 (75.8)	225,123 (58.4)	
University of Pennsylvania	67,314 (25.1)	26,929 (32.6)	4,623 (25.7)	3,398 (20.3)	102,264 (26.5)	
Sanford Health	56,553 (21.1)	328 (0.4)	586 (3.3)	649 (3.9)	58,116 (15.1)	

Note: Data are expressed as number (percentage) except as indicated. DBT = digital breast tomosynthesis; DM = digital mammography; IQR = interquartile range.

* Analysis of variance.

† Chi-square test.

‡ Gail model (5-year risk) or Tyrer–Cuzick (lifetime risk).

**Table 3.** Recall rates stratified by race and age for all women

		White			Black			Asian			Other			All Women		
		Unique Women (n = 268,309)			Unique Women (n = 82,522)			Unique Women (n = 17,961)			Unique Women (n =16,711)			Unique Women (n = 385,503)		
Age Categories	All Screens	DBT	DM	P	DBT	DM	P	DBT	DM	P	DBT	DM	P	DBT	DM	P

n (screens)		410,493	167,394		91,054	71,939		23,993	9,443		17,405	12,583		542,945	261,359	
40–44 y	79,480	13.54	16.00		13.09	17.28		15.05	18.00		12.15	13.28		13.49	16.17	
45–49 y	103,018	11.43	13.16		10.51	13.19		11.06	14.85		10.63	12.60		11.21	13.21	
50–59 y	251,854	8.76	9.40		8.55	10.49		10.06	12.22		7.60	8.78		8.74	9.76	
60–69 y	150,308	7.13	8.36		7.38	9.92		8.39	11.81		6.56	7.84		7.20	8.87	
70–79 y	219,644	6.64	7.72		6.72	8.77		7.64	9.29		6.55	7.31		6.69	8.07	
All		8.70	9.70	<.0001*	8.60	10.62	<.0001*	10.17	12.60	<.0001*	8.52	9.77	0.0001*	8.74	10.06	<.0001*

Note: DBT = digital breast tomosynthesis; DM = digital mammography.

* Indicates a significant difference between DBT and DM for that race at *p*<0.05, adjusted for age, breast density and institution.

**Table 4.** Recall rates stratified by race, age, and screening modality for women with only one observed screen

		White			Black			Asian			Other			All Women		
		Unique Women (n = 97,456)			Unique Women (n = 35,465)			Unique Women (n = 8,692)			Unique Women (n = 8,422)			Unique Women (n = 150,035)		
Age Categories	All Screens	DBT	DM	P	DBT	DM	P	DBT	DM	P	DBT	DM	P	DBT	DM	P

n (screens)		67,657	29,799		20,605	14,860		6,175	2,517		4,872	3,550		99,309	50,726	
40–44 y	26,040	19.35	23.44		18.31	24.00		22.64	25.17		17.66	18.18		19.26	23.14	
45–49 y	22,304	17.58	21.56		16.78	20.25		15.53	24.04		13.95	17.98		17.04	21.01	
50–59 y	46,174	15.37	17.93		13.95	18.21		15.57	20.92		12.52	14.62		14.95	17.92	
60–69 y	23,697	13.55	16.61		12.41	17.23		15.86	19.43		13.61	12.33		13.45	16.69	
70–79 y	31,820	12.30	14.47		11.95	14.76		14.45	16.61		9.92	12.78		12.25	14.58	
All		15.53	18.06	<.0001*	14.65	18.10	<.0001*	16.96	20.98	0.0003*	13.75	15.35	0.0698	15.35	18.03	<.0001*

Note: DBT = digital breast tomosynthesis; DM = digital mammography.

* Indicates a significant difference between DBT and DM for that race at *P* < .05, adjusted for age, breast density, and institution.

**Table 5.** Recall rates stratified by race, age, and screening modality for women with 2 or more observed screens

		White			Black			Asian			Other			All Women		
		Unique Women (n = 170,853)			Unique Women (n = 47,057)			Unique Women (n = 9,269)			Unique Women (n = 8,289)			Unique Women (n = 235,468)		
Age Categories	All Screens	DBT	DM	P	DBT	DM	P	DBT	DM	P	DBT	DM	P	DBT	DM	P

n (screens)		342,836	137,595		70,449	57,079		17,818	6,926		12,533	9,033		443,636	210,633	
40–44 y	53,440	10.95	13.16		9.31	13.92	<.0001*	10.18	13.96		8.00	10.51		10.54	13.16	
45–49 y	80,714	10.00	10.91		8.23	10.77		9.46	11.20		9.37	10.36		9.66	10.85	
50–59 y	205,680	7.49	7.58		6.99	8.37		8.23	9.26		5.90	6.70		7.39	7.80	
60–69 y	126,611	6.09	6.85		6.19	8.33		6.24	9.61		4.42	6.39		6.07	7.31	
70–79 y	187,824	5.84	6.49		5.76	7.54		5.95	6.83		5.51	5.31		5.82	6.79	
All		7.35	7.89	<.0001*	6.83	8.67	<.0001*	7.40	8.82	<.0001*	6.49	7.57	0.003*	7.26	8.14	<.0001*

Note: DBT = digital breast tomosynthesis; DM = digital mammography.

* Indicates a significant difference between DBT and DM for that race at *P* < .05, adjusted for age, breast density, and institution.

**Table 6.** Adjusted odds ratios for the effect of screening modality (digital breast tomosynthesis vs digital mammography) on recall, overall, and by race

	White			Black			Asian			Other			All Women		
	Unique Women (n = 268,309)			Unique Women (n = 82,522)			Unique Women (n = 17,961)			Unique Women (n = 16,711)			Unique Women (n = 385,503)		
		95% CI			95% CI			95% CI			95% CI			95% CI	
Age Category	OR	Lower	Upper	OR	Lower	Upper	OR	Lower	Upper	OR	Lower	Upper	OR	Lower	Upper

40–44 y*	0.819	0.775	0.866	0.706	0.641	0.778	0.833	0.696	0.997	0.882	0.734	1.059	0.805	0.770	0.842
45–49 y*	0.878	0.834	0.925	0.815	0.744	0.893	0.777	0.650	0.929	0.813	0.681	0.971	0.861	0.825	0.898
50–59 y*	0.958	0.923	0.994	0.866	0.814	0.922	0.832	0.722	0.959	0.826	0.705	0.967	0.924	0.897	0.953
60–69 y*	0.888	0.845	0.933	0.776	0.712	0.845	0.725	0.599	0.876	0.818	0.637	1.049	0.845	0.811	0.880
70–79 y*	0.892	0.854	0.931	0.840	0.783	0.901	0.894	0.746	1.073	0.914	0.728	1.148	0.874	0.844	0.905
All^†^	0.897	0.879	0.916	0.813	0.785	0.841	0.817	0.756	0.881	0.844	0.775	0.919	0.870	0.856	0.885

Note: CI = confidence interval; OR = odds ratio.

* By age group: OR adjusted for institution and breast density.

† All: OR adjusted for age, institution, and breast density.

**Table 7.** Cancer detection rates by modality, age and race

	DBT			DM			
	Number of Screens	Cancers Detected	Cancers Detected per 1,000 Screens	Number of Screens	Cancers Detected	Cancers Detected per 1,000 Screens	P

Total	318,311	1,507	4.73	181,065	832	4.60	.0011*
40–45 y	42,110	97	2.30	19,997	38	1.90	.9222
46–49 y	34,706	101	2.91	17,275	54	3.13	.6498
50–59 y	100,591	431	4.28	56,393	216	3.83	.0590
60–69 y	92,934	531	5.71	55,670	307	5.51	.0585
70–79 y	47,970	347	7.23	31,730	217	6.84	.0371*
White	245,480	1,175	4.79	114,879	531	4.62	.0016*
Black	50,552	247	4.89	50,440	240	4.76	.2083
Asian	13,347	61	4.57	6,666	33	4.95	.7884
Other	8,932	24	2.69	9,080	28	3.08	.6877

Note: Outcomes are based on tumor registry results only. Complete tumor registry data were available for a subset of the study period, and only recall data from that time period were included in this analysis. *P* values are for the comparison between DBT and DM for that level of each characteristic, adjusted for age category, institution, race, breast density, and number of screens when that is not the comparison being calculated (eg, the comparison between DBT and DM for 40–45 years is not adjusted for age). DBT = digital breast tomosynthesis; DM = digital mammography.

* *P* < .05.

**Table 8.** Positive predictive value 1 for cancer screening, by age, race, and number of screens

	DBT			DM				
	Number of Cancers Among Recalls	Number of Recalls	PPV1	Number of Cancers Among Recalls	Number of Recalls	PPV1	Difference in PPV1 (DBT-DM)	*P*

Total	1,507	28,510	5.29	832	18,682	4.45	0.83	<.0001*
40–44 y	71	4,708	1.51	32	2,612	1.23	0.28	.6383
45–49 y	127	4,930	2.58	60	2,882	2.08	0.49	.3099
50–59 y	431	9,011	4.78	216	5,670	3.81	0.97	.0121*
60–69 y	531	6,660	7.97	307	5,019	6.12	1.86	.0002*
70–79 y	347	3,201	10.84	217	2,499	8.68	2.16	.0040*
White	1,175	21,830	5.38	531	11,488	4.62	0.76	<.0001*
Black	247	4,508	5.48	240	5,427	4.42	1.06	.0033*
Asian	61	1,416	4.31	33	867	3.81	0.5	.4018
Other	24	756	3.17	28	900	3.11	0.06	.9113
1 screen	794	8,842	8.98	584	6,699	8.72	0.26	.759
≥2 screens	713	19,668	3.63	248	11,983	2.07	1.56	<.0001*
Almost entirely fatty (A)	49	835	5.87	34	727	4.68	1.19	.6090
Scattered fibroglandular densities (B)	621	10,460	5.94	397	8,053	4.93	1.01	.0022*
Heterogeneously dense (C)	707	13,986	5.06	348	8,477	4.11	0.95	<.0001*
Extremely dense (D)	128	3,124	4.1	53	1,385	3.83	0.27	.1064
Unknown	2	105	1.9	0	40	0	1.9	NA

Note: *P* values are for the comparison between DBT and DM for that level of each characteristic, adjusted for age category, institution, race, breast density, and number of screens when that is not the comparison being calculated (eg, the comparison between DBT and DM for 40–45 years is not adjusted for age). DBT = digital breast tomosynthesis; DM = digital mammography; PPV1 = positive predictive value.

* *P* < .05.

## TAKE-HOME POINTS

- This study demonstrated that relative to DM, the use of DBT for breast cancer screening is associated with improved patient screening metrics, including reductions in RRs (8.74 versus 10.06, adjusted *P* < .05) in parallel with increases in CDRs (4.73 versus 4.60, adjusted *P* < .05) and improved PPV1 (5.29 versus 4.45, adjusted *P* < .05) for nearly all ages groups and races.
- Racial differences in screening frequency and DBT utilization are likely to be rooted in social, economic, cultural, and educational disparities.
- Less frequent screening of Black women may indicate a need for improved access and educational strategies to emphasize the importance of regular screening.
- Expanded access to DBT and the opportunity for earlier diagnosis through improved cancer detection is especially relevant for Black women because of known later stages at diagnosis and therefore lower breast cancer survival rates for this racial subgroup.
- Organizations such as the US Preventive Services Task Force and the American College of Obstetricians and Gynecologists provide conflicting guidelines regarding screening frequency, but real-world data such as those from this study can be used in efforts for guideline improvement.

## References

[1] Bray F, Ferlay J, Soerjomataram I, Siegel RL, Torre LA, Jemal A. Global cancer statistics 2018: GLOBOCAN estimates of incidence and mortality worldwide for 36 cancers in 185 countries. CA Cancer J Clin 2018;68:394–424. 30207593 10.3322/caac.21492

[2] DeSantis CE, Ma J, Gaudet MM, Breast cancer statistics 2019. CA Cancer J Clin 2019;69:438–51. 31577379 10.3322/caac.21583

[3] Pisano ED, Gatsonis C, Hendrick E, Diagnostic performance of digital versus film mammography for breast-cancer screening. N Engl J Med 2005;353:1773–83. 16169887 10.1056/NEJMoa052911

[4] Hambly NM, McNicholas MM, Phelan N, Hargaden GC, O’Doherty A, Flanagan FL. Comparison of digital mammography and screen-film mammography in breast cancer screening: a review in the Irish breast screening program. AJR Am J Roentgenol 2009;193:1010–8. 19770323 10.2214/AJR.08.2157

[5] Kolb TM, Lichy J, Newhouse JH. Comparison of the performance of screening mammography, physical examination, and breast US and evaluation of factors that influence them: an analysis of 27,825 patient evaluations. Radiology 2002;225:165–75. 12355001 10.1148/radiol.2251011667

[6] Mandelson MT, Oestreicher N, Porter PL, Breast density as a predictor of mammographic detection: comparison of interval- and screen-detected cancers. J Natl Cancer Inst 2000;92:1081–7. 10880551 10.1093/jnci/92.13.1081

[7] Conant EF, Barlow WE, Herschorn SD, Association of digital breast tomosynthesis vs digital mammography with cancer detection and recall rates by age and breast density. JAMA Oncol 2019;5: 635–42. 30816931 10.1001/jamaoncol.2018.7078 PMC6512257

[8] Marinovich ML, Hunter KE, Macaskill P, Houssami N. Breast cancer screening using tomosynthesis or mammography: a metaanalysis of cancer detection and recall. J Natl Cancer Inst 2018;110:942–9. 30107542 10.1093/jnci/djy121

[9] Friedewald SM, Rafferty EA, Rose SL, Breast cancer screening using tomosynthesis in combination with digital mammography. JAMA 2014;311:2499–507. 25058084 10.1001/jama.2014.6095

[10] Conant EF, Zuckerman SP, McDonald ES, Five consecutive years of screening with digital breast tomosynthesis: outcomes by screening year and round. Radiology 2020;295:285–93. 32154771 10.1148/radiol.2020191751 PMC7193918

[11] American Cancer Society. Breast cancer facts & figures 2019–2020. Available at: https://www.cancer.org/content/dam/cancer-org/research/cancer-facts-and-statistics/breast-cancer-facts-and-figures/breast-cancerfacts-and-figures-2019-2020.pdf. Accessed January 26, 2021.

[12] Chlebowski RT, Chen Z, Anderson GL, Ethnicity and breast cancer: factors influencing differences in incidence and outcome. J Natl Cancer Inst 2005;97:439–48. 15770008 10.1093/jnci/dji064

[13] Silber JH, Rosenbaum PR, Clark AS, Characteristics associated with differences in survival among black and white women with breast cancer. JAMA 2013;310:389–97. 23917289 10.1001/jama.2013.8272

[14] Passmore SR, Williams-Parry KF, Casper E, Thomas SB. Message received: African American women and breast cancer screening. Health Promot Pract 2017;18:726–33. 28812929 10.1177/1524839917696714

[15] Sighoko D, Murphy A, Irizarry B, Rauscher G, Ferrans C, Ansell D. Changes in the racial disparity in breast cancer mortality in the ten US cities with the largest African American populations from 1999 to 2013; the reduction in breast cancer mortality disparity in Chicago. Cancer Causes Control 2017;28:563–8. 28275936 10.1007/s10552-017-0878-y PMC5400784

[16] Allen JD, Shelton RC, Harden E, Goldman RE. Follow-up of abnormal screening mammograms among low-income ethnically diverse women: findings from a qualitative study. Patient Educ Couns 2008;72:283–92. 18490127 10.1016/j.pec.2008.03.024

[17] Lannin DR, Mathews HF, Mitchell J, Swanson MS, Swanson FH, Edwards MS. Influence of socioeconomic and cultural factors on racial differences in late-stage presentation of breast cancer. JAMA 1998;279: 1801–7. 9628711 10.1001/jama.279.22.1801

[18] Miles RC, Onega T, Lee CI. Addressing potential health disparities in the adoption of advanced breast imaging technologies. Acad Radiol 25: 547–551. 29729855 10.1016/j.acra.2017.05.021 PMC6420779

[19] Richman IB, Hoag JR, Xu X, Adoption of digital breast tomosynthesis in clinical practice. JAMA Intern Med 2019;179: 1292–5. 31233086 10.1001/jamainternmed.2019.1058 PMC6593646

[20] American College of Radiology. ACR BI-RADS atlas: Breast Imaging Reporting and Data System. 5th ed. Reston, Virginia: American College of Radiology; 2013.

[21] del Carmen MG, Halpern EF, Kopans DB, Mammographic breast density and race. AJR Am J Roentgenol 2007;188:1147–50. 17377060 10.2214/AJR.06.0619

[22] Adams-Campbell LL, Makambi KH, Palmer JR, Rosenberg L. Diagnostic accuracy of the Gail model in the Black Women’s Health Study. Breast J 2007;13:332–6. 17593036 10.1111/j.1524-4741.2007.00439.x

[23] Rauscher GH, Allgood KL, Whitman S, Conant E. Disparities in screening mammography services by race/ethnicity and health insurance. J Womens Health (Larchmt) 2012;21:154–60. 21942866 10.1089/jwh.2010.2415 PMC3270049

[24] Harvey S, Gallagher AM, Nolan M, Hughes CM. Listening to women: expectations and experiences in breast imaging. J Womens Health (Larchmt) 2015;24:777–83. 26390380 10.1089/jwh.2015.29001.swh PMC4589306

[25] Bonafede MM, Kalra VB, Miller JD, Fajardo LL. Value analysis of digital breast tomosynthesis for breast cancer screening in a commercially-insured US population. Clinicoecon Outcomes Res 2015;7:53–63. 25624767 10.2147/CEOR.S76167 PMC4296908

[26] Alcusky M, Philpotts L, Bonafede M, Clarke J, Skoufalos A. The patient burden of screening mammography recall. J Womens Health (Larchmt) 2014;23(suppl 1):S11–9. 25247382 10.1089/jwh.2014.1511

[27] Samorani M, Blount LG. Machine learning and medical appointment scheduling: creating and perpetuating inequalities in access to health care. Am J Public Health 2020;110:440–1. 32159974 10.2105/AJPH.2020.305570 PMC7067080

[28] Jafri N, Ayyala R, Ozonoff A, Jordan-Gray J, Slanetz P. Screening mammography: does ethnicity influence patient preferences for higher recall rates given the potential for earlier detection of breast cancer? Radiology 2008;249:785–91. 18941163 10.1148/radiol.2493072176

